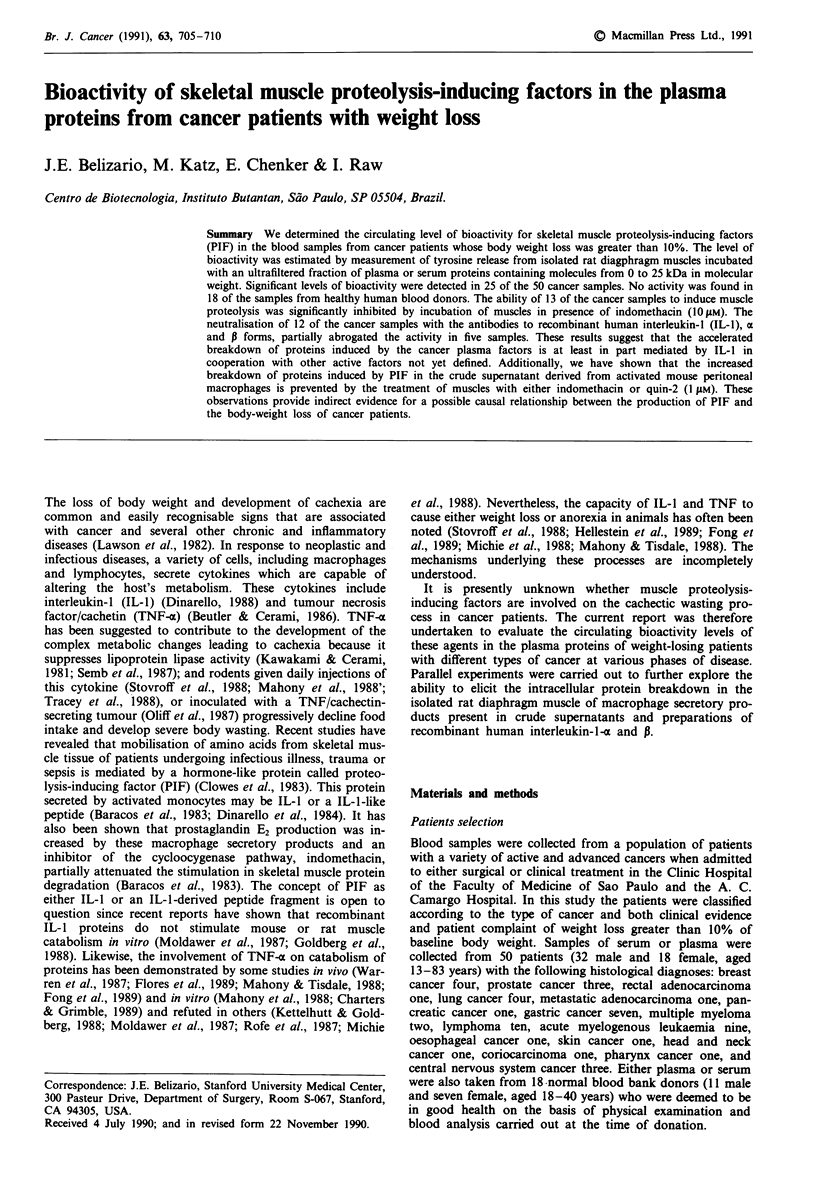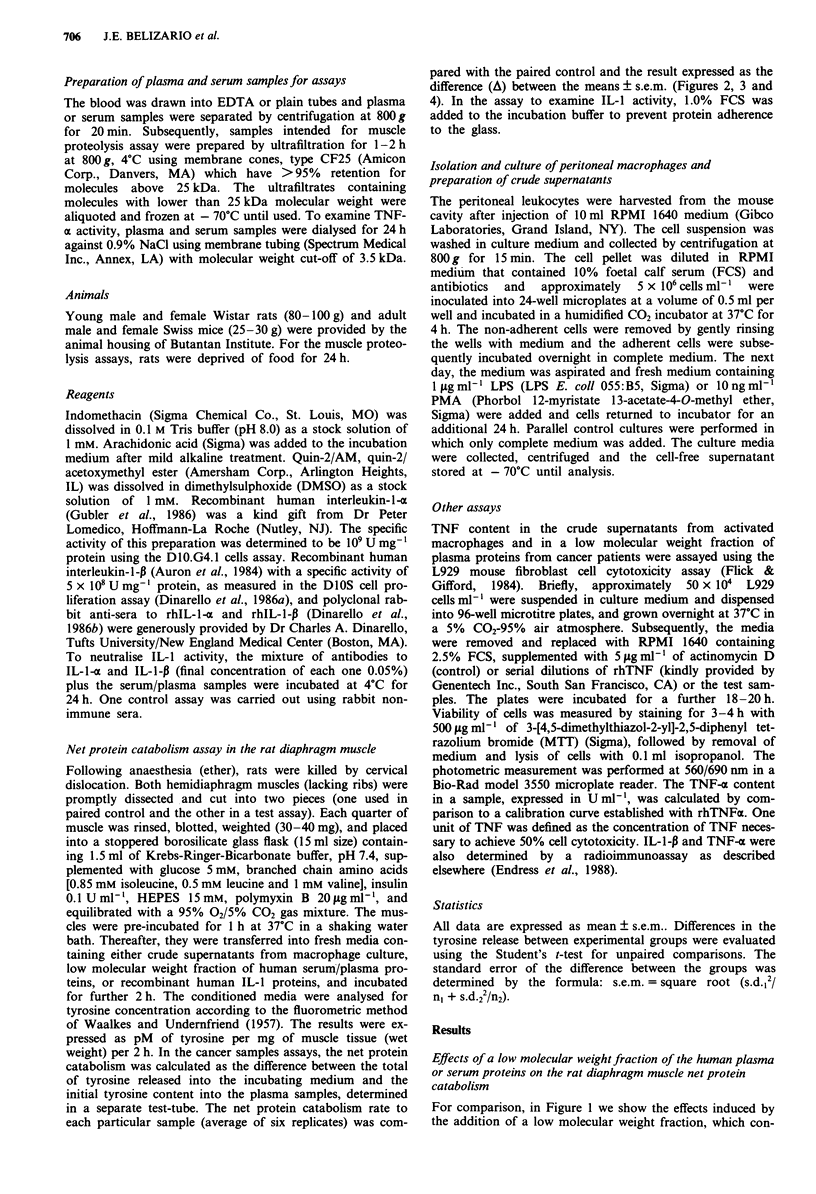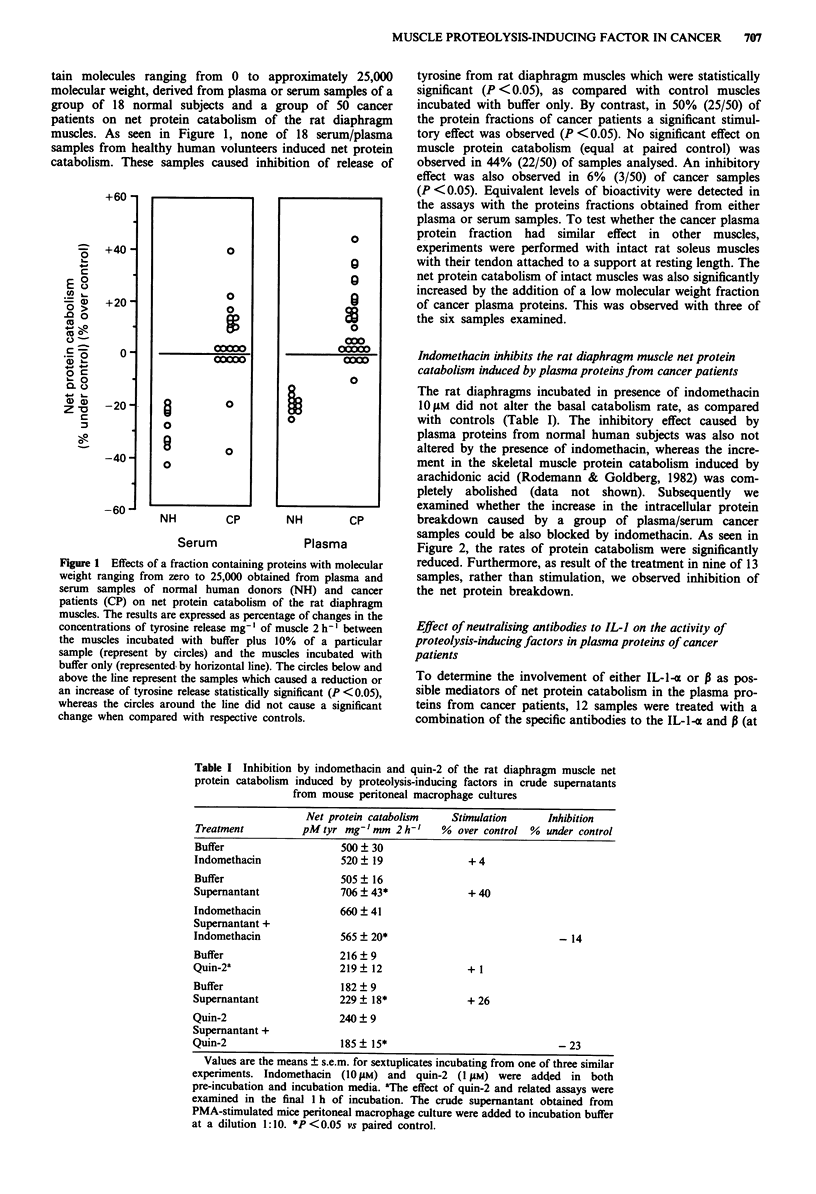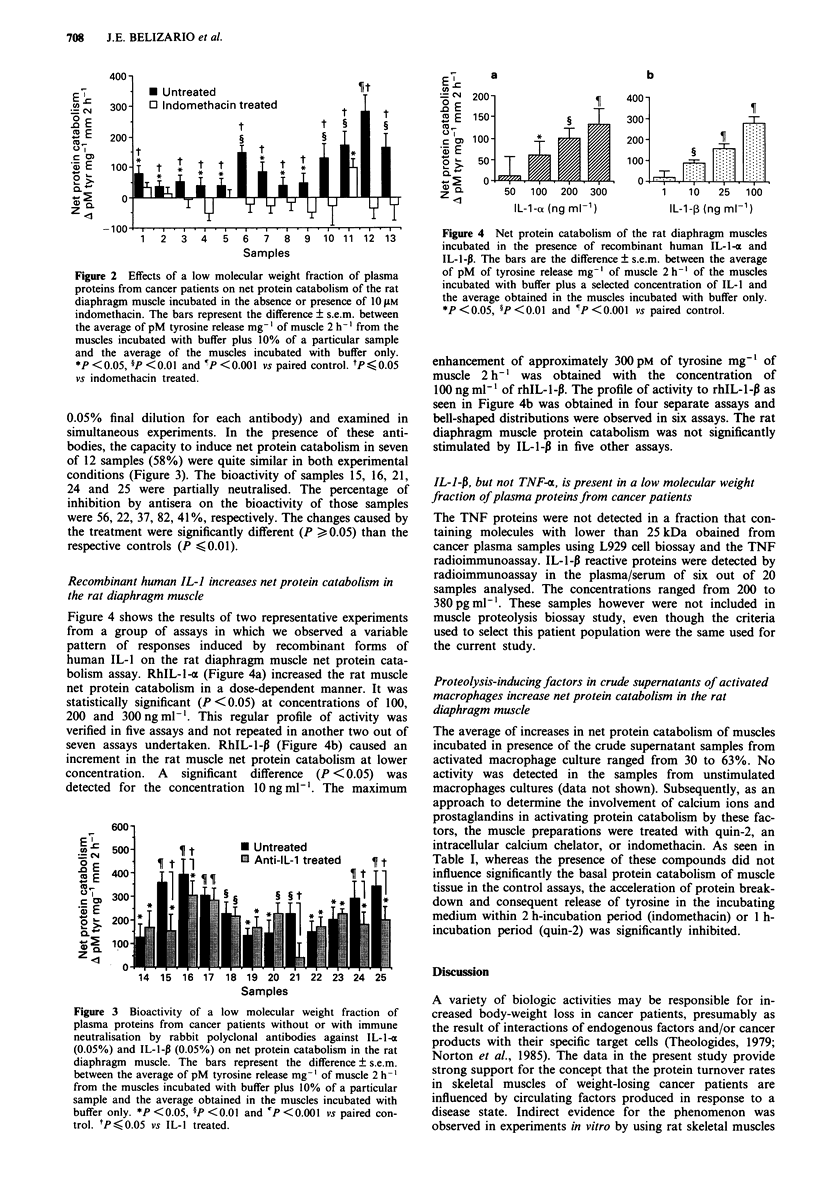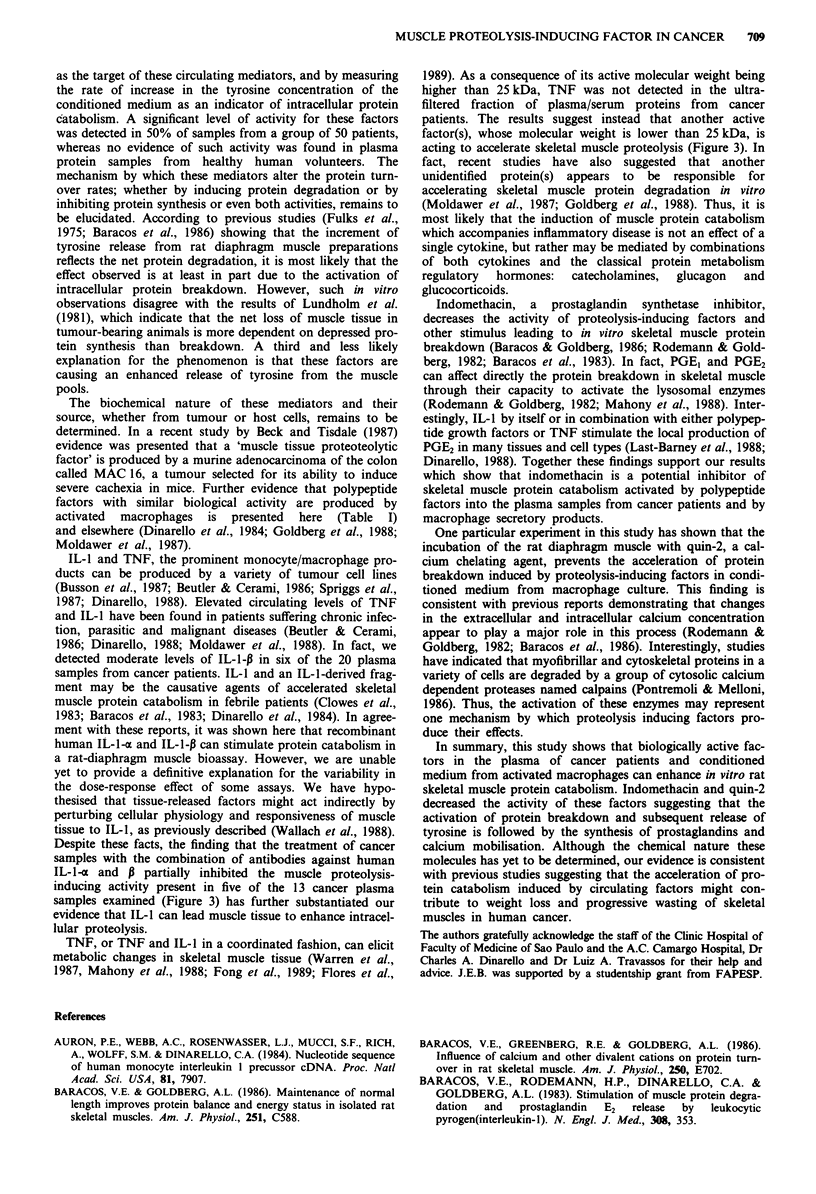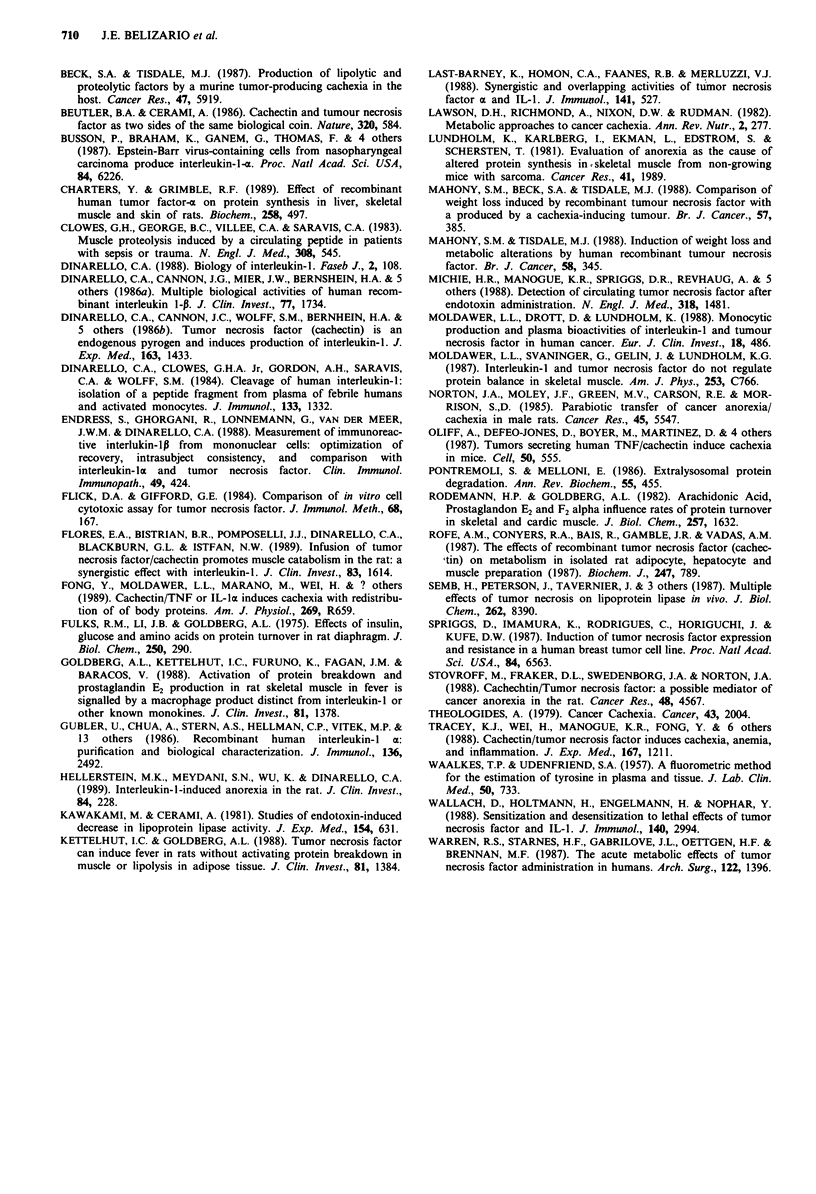# Bioactivity of skeletal muscle proteolysis-inducing factors in the plasma proteins from cancer patients with weight loss.

**DOI:** 10.1038/bjc.1991.159

**Published:** 1991-05

**Authors:** J. E. Belizario, M. Katz, E. Chenker, I. Raw

**Affiliations:** Centro de Biotecnologia, Instituto Butantan, São Paulo, Brazil.

## Abstract

We determined the circulating level of bioactivity for skeletal muscle proteolysis-inducing factors (PIF) in the blood samples from cancer patients whose body weight loss was greater than 10%. The level of bioactivity was estimated by measurement of tyrosine release from isolated 1at diaphragm muscles incubated with an ultrafiltered fraction of plasma or serum proteins containing molecules from 0 to 25 kDa in molecular weight. Significant levels of bioactivity were detected in 25 of the 50 cancer samples. No activity was found in 18 of the samples from healthy human blood donors. The ability of 13 of the cancer samples to induce muscle proteolysis was significantly inhibited by incubation of muscles in presence of indomethacin (10 microM). The neutralisation of 12 of the cancer samples with the antibodies to recombinant human interleukin-1 (IL-1), alpha and beta forms, partially abrogated the activity in five samples. These results suggest that the accelerated breakdown of proteins induced by the cancer plasma factors is at least in part mediated by IL-1 in cooperation with other active factors not yet defined. Additionally, we have shown that the increased breakdown of proteins induced by PIF in the crude supernatant derived from activated mouse peritoneal macrophages is prevented by the treatment of muscles with either indomethacin or quin-2 (1 microM). These observations provide indirect evidence for a possible causal relationship between the production of PIF and the body-weight loss of cancer patients.


Br. J. Cancer (1991), 63, 705–710 © Macmillan Press Ltd., 1991

### Bioactivity of skeletal muscle proteolysis-inducing factors in the plasma proteins from cancer patients with weight loss

J.E. Belizario, M. Katz, E. Chenker & I. Raw

*Centro de Biotecnologia, Instituto Butantan, São Paulo, SP 05504, Brazil.*

**Summary** We determined the circulating level of bioactivity for skeletal muscle proteolysis-inducing factors (PIF) in the blood samples from cancer patients whose body weight loss was greater than 10%. The level of bioactivity was estimated by measurement of tyrosine release from isolated rat diagphragm muscles incubated with an ultrafiltered fraction of plasma or serum proteins containing molecules from 0 to 25 kDa in molecular weight. Significant levels of bioactivity were detected in 25 of the 50 cancer samples. No activity was found in 18 of the samples from healthy human blood donors. The ability of 13 of the cancer samples to induce muscle proteolysis was significantly inhibited by incubation of muscles in presence of indomethacin (10 μM). The neutralisation of 12 of the cancer samples with the antibodies to recombinant human interleukin-1 (IL-1), α and β forms, partially abrogated the activity in five samples. These results suggest that the accelerated breakdown of proteins induced by the cancer plasma factors is at least in part mediated by IL-1 in cooperation with other active factors not yet defined. Additionally, we have shown that the increased breakdown of proteins induced by PIF in the crude supernatant derived from activated mouse peritoneal macrophages is prevented by the treatment of muscles with either indomethacin or quin-2 (1 μM). These observations provide indirect evidence for a possible causal relationship between the production of PIF and the body-weight loss of cancer patients.

The loss of body weight and development of cachexia are common and easily recognisable signs that are associated with cancer and several other chronic and inflammatory diseases (Lawson *et al.*, 1982). In response to neoplastic and infectious diseases, a variety of cells, including macrophages and lymphocytes, secrete cytokines which are capable of altering the host's metabolism. These cytokines include interleukin-1 (IL-1) (Dinarello, 1988) and tumour necrosis factor/cachetin (TNF-α) (Beutler & Cerami, 1986). TNF-α has been suggested to contribute to the development of the complex metabolic changes leading to cachexia because it suppresses lipoprotein lipase activity (Kawakami & Cerami, 1981; Semb *et al.*, 1987); and rodents given daily injections of this cytokine (Stovroff *et al.*, 1988; Mahony *et al.*, 1988'; Tracey *et al.*, 1988), or inoculated with a TNF/cachectin-secreting tumour (Oliff *et al.*, 1987) progressively decline food intake and develop severe body wasting. Recent studies have revealed that mobilisation of amino acids from skeletal muscle tissue of patients undergoing infectious illness, trauma or sepsis is mediated by a hormone-like protein called proteolysis-inducing factor (PIF) (Clowes *et al.*, 1983). This protein secreted by activated monocytes may be IL-1 or a IL-1-like peptide (Baracos *et al.*, 1983; Dinarello *et al.*, 1984). It has also been shown that prostaglandin $E_2$ production was increased by these macrophage secretory products and an inhibitor of the cycloocygenase pathway, indomethacin, partially attenuated the stimulation in skeletal muscle protein degradation (Baracos *et al.*, 1983). The concept of PIF as either IL-1 or an IL-1-derived peptide fragment is open to question since recent reports have shown that recombinant IL-1 proteins do not stimulate mouse or rat muscle catabolism *in vitro* (Moldawer *et al.*, 1987; Goldberg *et al.*, 1988). Likewise, the involvement of TNF-α on catabolism of proteins has been demonstrated by some studies *in vivo* (Warren *et al.*, 1987; Flores *et al.*, 1989; Mahony & Tisdale, 1988; Fong *et al.*, 1989) and *in vitro* (Mahony *et al.*, 1988; Charters & Grimble, 1989) and refuted in others (Kettelhutt & Goldberg, 1988; Moldawer *et al.*, 1987; Rofe *et al.*, 1987; Michie *et al.*, 1988). Nevertheless, the capacity of IL-1 and TNF to cause either weight loss or anorexia in animals has often been noted (Stovroff *et al.*, 1988; Hellestein *et al.*, 1989; Fong *et al.*, 1989; Michie *et al.*, 1988; Mahony & Tisdale, 1988). The mechanisms underlying these processes are incompletely understood.

It is presently unknown whether muscle proteolysis-inducing factors are involved on the cachectic wasting process in cancer patients. The current report was therefore undertaken to evaluate the circulating bioactivity levels of these agents in the plasma proteins of weight-losing patients with different types of cancer at various phases of disease. Parallel experiments were carried out to further explore the ability to elicit the intracellular protein breakdown in the isolated rat diaphragm muscle of macrophage secretory products present in crude supernatants and preparations of recombinant human interleukin-1-α and β.

### Materials and methods

### *Patients selection*

Blood samples were collected from a population of patients with a variety of active and advanced cancers when admitted to either surgical or clinical treatment in the Clinic Hospital of the Faculty of Medicine of Sao Paulo and the A. C. Camargo Hospital. In this study the patients were classified according to the type of cancer and both clinical evidence and patient complaint of weight loss greater than 10% of baseline body weight. Samples of serum or plasma were collected from 50 patients (32 male and 18 female, aged 13–83 years) with the following histological diagnoses: breast cancer four, prostate cancer three, rectal adenocarcinoma one, lung cancer four, metastatic adenocarcinoma one, pancreatic cancer one, gastric cancer seven, multiple myeloma two, lymphoma ten, acute myelogenous leukaemia nine, oesophageal cancer one, skin cancer one, head and neck cancer one, coriocarcinoma one, pharynx cancer one, and central nervous system cancer three. Either plasma or serum were also taken from 18 normal blood bank donors (11 male and seven female, aged 18–40 years) who were deemed to be in good health on the basis of physical examination and blood analysis carried out at the time of donation.

Correspondence: J.E. Belizario, Stanford University Medical Center, 300 Pasteur Drive, Department of Surgery, Room S-067, Stanford, CA 94305, USA.
Received 4 July 1990; and in revised form 22 November 1990.

*Preparation of plasma and serum samples for assays*

The blood was drawn into EDTA or plain tubes and plasma or serum samples were separated by centrifugation at 800 *g* for 20 min. Subsequently, samples intended for muscle proteolysis assay were prepared by ultrafiltration for 1–2 h at 800 *g*, 4°C using membrane cones, type CF25 (Amicon Corp., Danvers, MA) which have >95% retention for molecules above 25 kDa. The ultrafiltrates containing molecules with lower than 25 kDa molecular weight were aliquoted and frozen at – 70°C until used. To examine TNF-α activity, plasma and serum samples were dialysed for 24 h against 0.9% NaCl using membrane tubing (Spectrum Medical Inc., Annex, LA) with molecular weight cut-off of 3.5 kDa.

*Animals*

Young male and female Wistar rats (80–100 g) and adult male and female Swiss mice (25–30 g) were provided by the animal housing of Butantan Institute. For the muscle proteolysis assays, rats were deprived of food for 24 h.

*Reagents*

Indomethacin (Sigma Chemical Co., St. Louis, MO) was dissolved in 0.1 M Tris buffer (pH 8.0) as a stock solution of 1 mM. Arachidonic acid (Sigma) was added to the incubation medium after mild alkaline treatment. Quin-2/AM, quin-2/acetoxymethyl ester (Amersham Corp., Arlington Heights, IL) was dissolved in dimethylsulphoxide (DMSO) as a stock solution of 1 mM. Recombinant human interleukin-1-α (Gubler *et al.*, 1986) was a kind gift from Dr Peter Lomedico, Hoffmann-La Roche (Nutley, NJ). The specific activity of this preparation was determined to be $10^9$ U mg$^{-1}$ protein using the D10.G4.1 cells assay. Recombinant human interleukin-1-β (Auron *et al.*, 1984) with a specific activity of $5 \times 10^8$ U mg$^{-1}$ protein, as measured in the D10S cell proliferation assay (Dinarello *et al.*, 1986*a*), and polyclonal rabbit anti-sera to rhIL-1-α and rhIL-1-β (Dinarello *et al.*, 1986*b*) were generously provided by Dr Charles A. Dinarello, Tufts University/New England Medical Center (Boston, MA). To neutralise IL-1 activity, the mixture of antibodies to IL-1-α and IL-1-β (final concentration of each one 0.05%) plus the serum/plasma samples were incubated at 4°C for 24 h. One control assay was carried out using rabbit non-immune sera.

*Net protein catabolism assay in the rat diaphragm muscle*

Following anaesthesia (ether), rats were killed by cervical dislocation. Both hemidiaphragm muscles (lacking ribs) were promptly dissected and cut into two pieces (one used in paired control and the other in a test assay). Each quarter of muscle was rinsed, blotted, weighted (30–40 mg), and placed into a stoppered borosilicate glass flask (15 ml size) containing 1.5 ml of Krebs-Ringer-Bicarbonate buffer, pH 7.4, supplemented with glucose 5 mM, branched chain amino acids [0.85 mM isoleucine, 0.5 mM leucine and 1 mM valine], insulin 0.1 U ml$^{-1}$, HEPES 15 mM, polymyxin B 20 μg ml$^{-1}$, and equilibrated with a 95% $O_2$/5% $CO_2$ gas mixture. The muscles were pre-incubated for 1 h at 37°C in a shaking water bath. Thereafter, they were transferred into fresh media containing either crude supernatants from macrophage culture, low molecular weight fraction of human serum/plasma proteins, or recombinant human IL-1 proteins, and incubated for further 2 h. The conditioned media were analysed for tyrosine concentration according to the fluorometric method of Waalkes and Undernfriend (1957). The results were expressed as pM of tyrosine per mg of muscle tissue (wet weight) per 2 h. In the cancer samples assays, the net protein catabolism was calculated as the difference between the total of tyrosine released into the incubating medium and the initial tyrosine content into the plasma samples, determined in a separate test-tube. The net protein catabolism rate to each particular sample (average of six replicates) was compared with the paired control and the result expressed as the difference (Δ) between the means ± s.e.m. (Figures 2, 3 and 4). In the assay to examine IL-1 activity, 1.0% FCS was added to the incubation buffer to prevent protein adherence to the glass.

*Isolation and culture of peritoneal macrophages and preparation of crude supernatants*

The peritoneal leukocytes were harvested from the mouse cavity after injection of 10 ml RPMI 1640 medium (Gibco Laboratories, Grand Island, NY). The cell suspension was washed in culture medium and collected by centrifugation at 800 *g* for 15 min. The cell pellet was diluted in RPMI medium that contained 10% foetal calf serum (FCS) and antibiotics and approximately $5 \times 10^6$ cells ml$^{-1}$ were inoculated into 24-well microplates at a volume of 0.5 ml per well and incubated in a humidified $CO_2$ incubator at 37°C for 4 h. The non-adherent cells were removed by gently rinsing the wells with medium and the adherent cells were subsequently incubated overnight in complete medium. The next day, the medium was aspirated and fresh medium containing 1 μg ml$^{-1}$ LPS (LPS *E. coll* 055:B5, Sigma) or 10 ng ml$^{-1}$ PMA (Phorbol 12-myristate 13-acetate-4-*O*-methyl ether, Sigma) were added and cells returned to incubator for an additional 24 h. Parallel control cultures were performed in which only complete medium was added. The culture media were collected, centrifuged and the cell-free supernatant stored at – 70°C until analysis.

*Other assays*

TNF content in the crude supernatants from activated macrophages and in a low molecular weight fraction of plasma proteins from cancer patients were assayed using the L929 mouse fibroblast cell cytotoxicity assay (Flick & Gifford, 1984). Briefly, approximately $50 \times 10^4$ L929 cells ml$^{-1}$ were suspended in culture medium and dispensed into 96-well microtitre plates, and grown overnight at 37°C in a 5% $CO_2$-95% air atmosphere. Subsequently, the media were removed and replaced with RPMI 1640 containing 2.5% FCS, supplemented with 5 μg ml$^{-1}$ of actinomycin D (control) or serial dilutions of rhTNF (kindly provided by Genentech Inc., South San Francisco, CA) or the test samples. The plates were incubated for a further 18–20 h. Viability of cells was measured by staining for 3–4 h with 500 μg ml$^{-1}$ of 3-[4,5-dimethylthiazol-2-yl]-2,5-diphenyl tetrazolium bromide (MTT) (Sigma), followed by removal of medium and lysis of cells with 0.1 ml isopropanol. The photometric measurement was performed at 560/690 nm in a Bio-Rad model 3550 microplate reader. The TNF-α content in a sample, expressed in U ml$^{-1}$, was calculated by comparison to a calibration curve established with rhTNFα. One unit of TNF was defined as the concentration of TNF necessary to achieve 50% cell cytotoxicity. IL-1-β and TNF-α were also determined by a radioimmunoassay as described elsewhere (Endress *et al.*, 1988).

*Statistics*

All data are expressed as mean ± s.e.m.. Differences in the tyrosine release between experimental groups were evaluated using the Student's *t*-test for unpaired comparisons. The standard error of the difference between the groups was determined by the formula: s.e.m. = square root $(s.d._1^2/n_1 + s.d._2^2/n_2)$.

### Results

*Effects of a low molecular weight fraction of the human plasma or serum proteins on the rat diaphragm muscle net protein catabolism*

For comparison, in Figure 1 we show the effects induced by the addition of a low molecular weight fraction, which contain molecules ranging from 0 to approximately 25,000 molecular weight, derived from plasma or serum samples of a group of 18 normal subjects and a group of 50 cancer patients on net protein catabolism of the rat diaphragm muscles. As seen in Figure 1, none of 18 serum/plasma samples from healthy human volunteers induced net protein catabolism. These samples caused inhibition of release of tyrosine from rat diaphragm muscles which were statistically significant ($P < 0.05$), as compared with control muscles incubated with buffer only. By contrast, in 50% (25/50) of the protein fractions of cancer patients a significant stimultory effect was observed ($P < 0.05$). No significant effect on muscle protein catabolism (equal at paired control) was observed in 44% (22/50) of samples analysed. An inhibitory effect was also observed in 6% (3/50) of cancer samples ($P < 0.05$). Equivalent levels of bioactivity were detected in the assays with the proteins fractions obtained from either plasma or serum samples. To test whether the cancer plasma protein fraction had similar effect in other muscles, experiments were performed with intact rat soleus muscles with their tendon attached to a support at resting length. The net protein catabolism of intact muscles was also significantly increased by the addition of a low molecular weight fraction of cancer plasma proteins. This was observed with three of the six samples examined.

**Figure 1** Effects of a fraction containing proteins with molecular weight ranging from zero to 25,000 obtained from plasma and serum samples of normal human donors (NH) and cancer patients (CP) on net protein catabolism of the rat diaphragm muscles. The results are expressed as percentage of changes in the concentrations of tyrosine release $mg^{-1}$ of muscle $2 h^{-1}$ between the muscles incubated with buffer plus 10% of a particular sample (represent by circles) and the muscles incubated with buffer only (represented by horizontal line). The circles below and above the line represent the samples which caused a reduction or an increase of tyrosine release statistically significant ($P < 0.05$), whereas the circles around the line did not cause a significant change when compared with respective controls.

*Indomethacin inhibits the rat diaphragm muscle net protein catabolism induced by plasma proteins from cancer patients*

The rat diaphragms incubated in presence of indomethacin 10 μM did not alter the basal catabolism rate, as compared with controls (Table I). The inhibitory effect caused by plasma proteins from normal human subjects was also not altered by the presence of indomethacin, whereas the increment in the skeletal muscle protein catabolism induced by arachidonic acid (Rodemann & Goldberg, 1982) was completely abolished (data not shown). Subsequently we examined whether the increase in the intracellular protein breakdown caused by a group of plasma/serum cancer samples could be also blocked by indomethacin. As seen in Figure 2, the rates of protein catabolism were significantly reduced. Furthermore, as result of the treatment in nine of 13 samples, rather than stimulation, we observed inhibition of the net protein breakdown.

*Effect of neutralising antibodies to IL-1 on the activity of proteolysis-inducing factors in plasma proteins of cancer patients*

To determine the involvement of either IL-1-α or β as possible mediators of net protein catabolism in the plasma proteins from cancer patients, 12 samples were treated with a combination of the specific antibodies to the IL-1-α and β (at

**Table I** Inhibition by indomethacin and quin-2 of the rat diaphragm muscle net protein catabolism induced by proteolysis-inducing factors in crude supernatants from mouse peritoneal macrophage cultures

| Treatment | Net protein catabolism pM tyr $mg^{-1}$ mm $2 h^{-1}$ | Stimulation % over control | Inhibition % under control |
|---|---|---|---|
| Buffer | 500 ± 30 | | |
| Indomethacin | 520 ± 19 | + 4 | |
| Buffer | 505 ± 16 | | |
| Supernantant | 706 ± 43* | + 40 | |
| Indomethacin | 660 ± 41 | | |
| Supernantant + Indomethacin | 565 ± 20* | | − 14 |
| Buffer | 216 ± 9 | | |
| Quin-2[a] | 219 ± 12 | + 1 | |
| Buffer | 182 ± 9 | | |
| Supernantant | 229 ± 18* | + 26 | |
| Quin-2 | 240 ± 9 | | |
| Supernantant + Quin-2 | 185 ± 15* | | − 23 |

Values are the means ± s.e.m. for sextuplicates incubating from one of three similar experiments. Indomethacin (10 μM) and quin-2 (1 μM) were added in both pre-incubation and incubation media. [a]The effect of quin-2 and related assays were examined in the final 1 h of incubation. The crude supernatant obtained from PMA-stimulated mice peritoneal macrophage culture were added to incubation buffer at a dilution 1:10. *$P < 0.05$ *vs* paired control.

**Figure 2** Effects of a low molecular weight fraction of plasma proteins from cancer patients on net protein catabolism of the rat diaphragm muscle incubated in the absence or presence of 10 μM indomethacin. The bars represent the difference ± s.e.m. between the average of pM tyrosine release $mg^{-1}$ of muscle $2\ h^{-1}$ from the muscles incubated with buffer plus 10% of a particular sample and the average of the muscles incubated with buffer only. *$P < 0.05$, §$P < 0.01$ and ¶$P < 0.001$ *vs* paired control. †$P \leqslant 0.05$ *vs* indomethacin treated.

0.05% final dilution for each antibody) and examined in simultaneous experiments. In the presence of these antibodies, the capacity to induce net protein catabolism in seven of 12 samples (58%) were quite similar in both experimental conditions (Figure 3). The bioactivity of samples 15, 16, 21, 24 and 25 were partially neutralised. The percentage of inhibition by antisera on the bioactivity of those samples were 56, 22, 37, 82, 41%, respectively. The changes caused by the treatment were significantly different ($P \geqslant 0.05$) than the respective controls ($P \leqslant 0.01$).

*Recombinant human IL-1 increases net protein catabolism in the rat diaphragm muscle*

Figure 4 shows the results of two representative experiments from a group of assays in which we observed a variable pattern of responses induced by recombinant forms of human IL-1 on the rat diaphragm muscle net protein catabolism assay. RhIL-1-α (Figure 4a) increased the rat muscle net protein catabolism in a dose-dependent manner. It was statistically significant ($P < 0.05$) at concentrations of 100, 200 and 300 ng $ml^{-1}$. This regular profile of activity was verified in five assays and not repeated in another two out of seven assays undertaken. RhIL-1-β (Figure 4b) caused an increment in the rat muscle net protein catabolism at lower concentration. A significant difference ($P < 0.05$) was detected for the concentration 10 ng $ml^{-1}$. The maximum

**Figure 3** Bioactivity of a low molecular weight fraction of plasma proteins from cancer patients without or with immune neutralisation by rabbit polyclonal antibodies against IL-1-α (0.05%) and IL-1-β (0.05%) on net protein catabolism in the rat diaphragm muscle. The bars represent the difference ± s.e.m. between the average of pM tyrosine release $mg^{-1}$ of muscle $2\ h^{-1}$ from the muscles incubated with buffer plus 10% of a particular sample and the average obtained in the muscles incubated with buffer only. *$P < 0.05$, §$P < 0.01$ and ¶$P < 0.001$ *vs* paired control. †$P \leqslant 0.05$ *vs* IL-1 treated.

**Figure 4** Net protein catabolism of the rat diaphragm muscles incubated in the presence of recombinant human IL-1-α and IL-1-β. The bars are the difference ± s.e.m. between the average of pM of tyrosine release $mg^{-1}$ of muscle $2\ h^{-1}$ of the muscles incubated with buffer plus a selected concentration of IL-1 and the average obtained in the muscles incubated with buffer only. *$P < 0.05$, §$P < 0.01$ and ¶$P < 0.001$ *vs* paired control.

enhancement of approximately 300 pM of tyrosine $mg^{-1}$ of muscle $2\ h^{-1}$ was obtained with the concentration of 100 ng $ml^{-1}$ of rhIL-1-β. The profile of activity to rhIL-1-β as seen in Figure 4b was obtained in four separate assays and bell-shaped distributions were observed in six assays. The rat diaphragm muscle protein catabolism was not significantly stimulated by IL-1-β in five other assays.

*IL-1-β, but not TNF-α, is present in a low molecular weight fraction of plasma proteins from cancer patients*

The TNF proteins were not detected in a fraction that containing molecules with lower than 25 kDa obained from cancer plasma samples using L929 cell biossay and the TNF radioimmunoassay. IL-1-β reactive proteins were detected by radioimmunoassay in the plasma/serum of six out of 20 samples analysed. The concentrations ranged from 200 to 380 pg $ml^{-1}$. These samples however were not included in muscle proteolysis biossay study, even though the criteria used to select this patient population were the same used for the current study.

*Proteolysis-inducing factors in crude supernatants of activated macrophages increase net protein catabolism in the rat diaphragm muscle*

The average of increases in net protein catabolism of muscles incubated in presence of the crude supernatant samples from activated macrophage culture ranged from 30 to 63%. No activity was detected in the samples from unstimulated macrophages cultures (data not shown). Subsequently, as an approach to determine the involvement of calcium ions and prostaglandins in activating protein catabolism by these factors, the muscle preparations were treated with quin-2, an intracellular calcium chelator, or indomethacin. As seen in Table I, whereas the presence of these compounds did not influence significantly the basal protein catabolism of muscle tissue in the control assays, the acceleration of protein breakdown and consequent release of tyrosine in the incubating medium within 2 h-incubation period (indomethacin) or 1 h-incubation period (quin-2) was significantly inhibited.

### Discussion

A variety of biologic activities may be responsible for increased body-weight loss in cancer patients, presumably as the result of interactions of endogenous factors and/or cancer products with their specific target cells (Theologides, 1979; Norton *et al.*, 1985). The data in the present study provide strong support for the concept that the protein turnover rates in skeletal muscles of weight-losing cancer patients are influenced by circulating factors produced in response to a disease state. Indirect evidence for the phenomenon was observed in experiments *in vitro* by using rat skeletal muscles as the target of these circulating mediators, and by measuring the rate of increase in the tyrosine concentration of the conditioned medium as an indicator of intracellular protein catabolism. A significant level of activity for these factors was detected in 50% of samples from a group of 50 patients, whereas no evidence of such activity was found in plasma protein samples from healthy human volunteers. The mechanism by which these mediators alter the protein turnover rates; whether by inducing protein degradation or by inhibiting protein synthesis or even both activities, remains to be elucidated. According to previous studies (Fulks *et al.*, 1975; Baracos *et al.*, 1986) showing that the increment of tyrosine release from rat diaphragm muscle preparations reflects the net protein degradation, it is most likely that the effect observed is at least in part due to the activation of intracellular protein breakdown. However, such *in vitro* observations disagree with the results of Lundholm *et al.* (1981), which indicate that the net loss of muscle tissue in tumour-bearing animals is more dependent on depressed protein synthesis than breakdown. A third and less likely explanation for the phenomenon is that these factors are causing an enhanced release of tyrosine from the muscle pools.

The biochemical nature of these mediators and their source, whether from tumour or host cells, remains to be determined. In a recent study by Beck and Tisdale (1987) evidence was presented that a 'muscle tissue proteoteolytic factor' is produced by a murine adenocarcinoma of the colon called MAC 16, a tumour selected for its ability to induce severe cachexia in mice. Further evidence that polypeptide factors with similar biological activity are produced by activated macrophages is presented here (Table I) and elsewhere (Dinarello *et al.*, 1984; Goldberg *et al.*, 1988; Moldawer *et al.*, 1987).

IL-1 and TNF, the prominent monocyte/macrophage products can be produced by a variety of tumour cell lines (Busson *et al.*, 1987; Beutler & Cerami, 1986; Spriggs *et al.*, 1987; Dinarello, 1988). Elevated circulating levels of TNF and IL-1 have been found in patients suffering chronic infection, parasitic and malignant diseases (Beutler & Cerami, 1986; Dinarello, 1988; Moldawer *et al.*, 1988). In fact, we detected moderate levels of IL-1-$\beta$ in six of the 20 plasma samples from cancer patients. IL-1 and an IL-1-derived fragment may be the causative agents of accelerated skeletal muscle protein catabolism in febrile patients (Clowes *et al.*, 1983; Baracos *et al.*, 1983; Dinarello *et al.*, 1984). In agreement with these reports, it was shown here that recombinant human IL-1-$\alpha$ and IL-1-$\beta$ can stimulate protein catabolism in a rat-diaphragm muscle bioassay. However, we are unable yet to provide a definitive explanation for the variability in the dose-response effect of some assays. We have hypothesised that tissue-released factors might act indirectly by perturbing cellular physiology and responsiveness of muscle tissue to IL-1, as previously described (Wallach *et al.*, 1988). Despite these facts, the finding that the treatment of cancer samples with the combination of antibodies against human IL-1-$\alpha$ and $\beta$ partially inhibited the muscle proteolysis-inducing activity present in five of the 13 cancer plasma samples examined (Figure 3) has further substantiated our evidence that IL-1 can lead muscle tissue to enhance intracellular proteolysis.

TNF, or TNF and IL-1 in a coordinated fashion, can elicit metabolic changes in skeletal muscle tissue (Warren *et al.*, 1987, Mahony *et al.*, 1988; Fong *et al.*, 1989; Flores *et al.*, 1989). As a consequence of its active molecular weight being higher than 25 kDa, TNF was not detected in the ultrafiltered fraction of plasma/serum proteins from cancer patients. The results suggest instead that another active factor(s), whose molecular weight is lower than 25 kDa, is acting to accelerate skeletal muscle proteolysis (Figure 3). In fact, recent studies have also suggested that another unidentified protein(s) appears to be responsible for accelerating skeletal muscle protein degradation *in vitro* (Moldawer *et al.*, 1987; Goldberg *et al.*, 1988). Thus, it is most likely that the induction of muscle protein catabolism which accompanies inflammatory disease is not an effect of a single cytokine, but rather may be mediated by combinations of both cytokines and the classical protein metabolism regulatory hormones: catecholamines, glucagon and glucocorticoids.

Indomethacin, a prostaglandin synthetase inhibitor, decreases the activity of proteolysis-inducing factors and other stimulus leading to *in vitro* skeletal muscle protein breakdown (Baracos & Goldberg, 1986; Rodemann & Goldberg, 1982; Baracos *et al.*, 1983). In fact, $PGE_1$ and $PGE_2$ can affect directly the protein breakdown in skeletal muscle through their capacity to activate the lysosomal enzymes (Rodemann & Goldberg, 1982; Mahony *et al.*, 1988). Interestingly, IL-1 by itself or in combination with either polypeptide growth factors or TNF stimulate the local production of $PGE_2$ in many tissues and cell types (Last-Barney *et al.*, 1988; Dinarello, 1988). Together these findings support our results which show that indomethacin is a potential inhibitor of skeletal muscle protein catabolism activated by polypeptide factors into the plasma samples from cancer patients and by macrophage secretory products.

One particular experiment in this study has shown that the incubation of the rat diaphragm muscle with quin-2, a calcium chelating agent, prevents the acceleration of protein breakdown induced by proteolysis-inducing factors in conditioned medium from macrophage culture. This finding is consistent with previous reports demonstrating that changes in the extracellular and intracellular calcium concentration appear to play a major role in this process (Rodemann & Goldberg, 1982; Baracos *et al.*, 1986). Interestingly, studies have indicated that myofibrillar and cytoskeletal proteins in a variety of cells are degraded by a group of cytosolic calcium dependent proteases named calpains (Pontremoli & Melloni, 1986). Thus, the activation of these enzymes may represent one mechanism by which proteolysis inducing factors produce their effects.

In summary, this study shows that biologically active factors in the plasma of cancer patients and conditioned medium from activated macrophages can enhance *in vitro* rat skeletal muscle protein catabolism. Indomethacin and quin-2 decreased the activity of these factors suggesting that the activation of protein breakdown and subsequent release of tyrosine is followed by the synthesis of prostaglandins and calcium mobilisation. Although the chemical nature these molecules has yet to be determined, our evidence is consistent with previous studies suggesting that the acceleration of protein catabolism induced by circulating factors might contribute to weight loss and progressive wasting of skeletal muscles in human cancer.

The authors gratefully acknowledge the staff of the Clinic Hospital of Faculty of Medicine of Sao Paulo and the A.C. Camargo Hospital, Dr Charles A. Dinarello and Dr Luiz A. Travassos for their help and advice. J.E.B. was supported by a studentship grant from FAPESP.

### References

AURON, P.E., WEBB, A.C., ROSENWASSER, L.J., MUCCI, S.F., RICH, A., WOLFF, S.M. & DINARELLO, C.A. (1984). Nucleotide sequence of human monocyte interleukin 1 precussor cDNA. *Proc. Natl Acad. Sci. USA*, **81**, 7907.

BARACOS, V.E. & GOLDBERG, A.L. (1986). Maintenance of normal length improves protein balance and energy status in isolated rat skeletal muscles. *Am. J. Physiol.*, **251**, C588.

BARACOS, V.E., GREENBERG, R.E. & GOLDBERG, A.L. (1986). Influence of calcium and other divalent cations on protein turnover in rat skeletal muscle. *Am. J. Physiol.*, **250**, E702.

BARACOS, V.E., RODEMANN, H.P., DINARELLO, C.A. & GOLDBERG, A.L. (1983). Stimulation of muscle protein degradation and prostaglandin $E_2$ release by leukocytic pyrogen(interleukin-1). *N. Engl. J. Med.*, **308**, 353.

BECK, S.A. & TISDALE, M.J. (1987). Production of lipolytic and proteolytic factors by a murine tumor-producing cachexia in the host. *Cancer Res.*, **47**, 5919.

BEUTLER, B.A. & CERAMI, A. (1986). Cachectin and tumour necrosis factor as two sides of the same biological coin. *Nature*, **320**, 584.

BUSSON, P., BRAHAM, K., GANEM, G., THOMAS, F. & 4 others (1987). Epstein-Barr virus-containing cells from nasopharyngeal carcinoma produce interleukin-1-α. *Proc. Natl Acad. Sci. USA*, **84**, 6226.

CHARTERS, Y. & GRIMBLE, R.F. (1989). Effect of recombinant human tumor factor-α on protein synthesis in liver, skeletal muscle and skin of rats. *Biochem.*, **258**, 497.

CLOWES, G.H., GEORGE, B.C., VILLEE, C.A. & SARAVIS, C.A. (1983). Muscle proteolysis induced by a circulating peptide in patients with sepsis or trauma. *N. Engl. J. Med.*, **308**, 545.

DINARELLO, C.A. (1988). Biology of interleukin-1. *Faseb J.*, **2**, 108.

DINARELLO, C.A., CANNON, J.G., MIER, J.W., BERNSHEIN, H.A. & 5 others (1986*a*). Multiple biological activities of human recombinant interleukin 1-β. *J. Clin. Invest.*, **77**, 1734.

DINARELLO, C.A., CANNON, J.C., WOLFF, S.M., BERNHEIN, H.A. & 5 others (1986*b*). Tumor necrosis factor (cachectin) is an endogenous pyrogen and induces production of interleukin-1. *J. Exp. Med.*, **163**, 1433.

DINARELLO, C.A., CLOWES, G.H.A. Jr, GORDON, A.H., SARAVIS, C.A. & WOLFF, S.M. (1984). Cleavage of human interleukin-1: isolation of a peptide fragment from plasma of febrile humans and activated monocytes. *J. Immunol.*, **133**, 1332.

ENDRESS, S., GHORGANI, R., LONNEMANN, G., VAN DER MEER, J.W.M. & DINARELLO, C.A. (1988). Measurement of immunoreactive interlukin-1β from mononuclear cells: optimization of recovery, intrasubject consistency, and comparison with interleukin-1α and tumor necrosis factor. *Clin. Immunol. Immunopath.*, **49**, 424.

FLICK, D.A. & GIFFORD, G.E. (1984). Comparison of *in vitro* cell cytotoxic assay for tumor necrosis factor. *J. Immunol. Meth.*, **68**, 167.

FLORES, E.A., BISTRIAN, B.R., POMPOSELLI, J.J., DINARELLO, C.A., BLACKBURN, G.L. & ISTFAN, N.W. (1989). Infusion of tumor necrosis factor/cachectin promotes muscle catabolism in the rat: a synergistic effect with interleukin-1. *J. Clin. Invest.*, **83**, 1614.

FONG, Y., MOLDAWER, L.L., MARANO, M., WEI, H. & ? others (1989). Cachectin/TNF or IL-1α induces cachexia with redistribution of of body proteins. *Am. J. Physiol.*, **269**, R659.

FULKS, R.M., LI, J.B. & GOLDBERG, A.L. (1975). Effects of insulin, glucose and amino acids on protein turnover in rat diaphragm. *J. Biol. Chem.*, **250**, 290.

GOLDBERG, A.L., KETTELHUT, I.C., FURUNO, K., FAGAN, J.M. & BARACOS, V. (1988). Activation of protein breakdown and prostaglandin $E_2$ production in rat skeletal muscle in fever is signalled by a macrophage product distinct from interleukin-1 or other known monokines. *J. Clin. Invest.*, **81**, 1378.

GUBLER, U., CHUA, A., STERN, A.S., HELLMAN, C.P., VITEK, M.P. & 13 others (1986). Recombinant human interleukin-1 α: purification and biological characterization. *J. Immunol.*, **136**, 2492.

HELLERSTEIN, M.K., MEYDANI, S.N., WU, K. & DINARELLO, C.A. (1989). Interleukin-1-induced anorexia in the rat. *J. Clin. Invest.*, **84**, 228.

KAWAKAMI, M. & CERAMI, A. (1981). Studies of endotoxin-induced decrease in lipoprotein lipase activity. *J. Exp. Med.*, **154**, 631.

KETTELHUT, I.C. & GOLDBERG, A.L. (1988). Tumor necrosis factor can induce fever in rats without activating protein breakdown in muscle or lipolysis in adipose tissue. *J. Clin. Invest.*, **81**, 1384.

LAST-BARNEY, K., HOMON, C.A., FAANES, R.B. & MERLUZZI, V.J. (1988). Synergistic and overlapping activities of tumor necrosis factor α and IL-1. *J. Immunol.*, **141**, 527.

LAWSON, D.H., RICHMOND, A., NIXON, D.W. & RUDMAN. (1982). Metabolic approaches to cancer cachexia. *Ann. Rev. Nutr.*, **2**, 277.

LUNDHOLM, K., KARLBERG, I., EKMAN, L., EDSTROM, S. & SCHERSTEN, T. (1981). Evaluation of anorexia as the cause of altered protein synthesis in skeletal muscle from non-growing mice with sarcoma. *Cancer Res.*, **41**, 1989.

MAHONY, S.M., BECK, S.A. & TISDALE, M.J. (1988). Comparison of weight loss induced by recombinant tumour necrosis factor with a produced by a cachexia-inducing tumour. *Br. J. Cancer.*, **57**, 385.

MAHONY, S.M. & TISDALE, M.J. (1988). Induction of weight loss and metabolic alterations by human recombinant tumour necrosis factor. *Br. J. Cancer*, **58**, 345.

MICHIE, H.R., MANOGUE, K.R., SPRIGGS, D.R., REVHAUG, A. & 5 others (1988). Detection of circulating tumor necrosis factor after endotoxin administration. *N. Engl. J. Med.*, **318**, 1481.

MOLDAWER, L.L., DROTT, D. & LUNDHOLM, K. (1988). Monocytic production and plasma bioactivities of interleukin-1 and tumour necrosis factor in human cancer. *Eur. J. Clin. Invest.*, **18**, 486.

MOLDAWER, L.L., SVANINGER, G., GELIN, J. & LUNDHOLM, K.G. (1987). Interleukin-1 and tumor necrosis factor do not regulate protein balance in skeletal muscle. *Am. J. Phys.*, **253**, C766.

NORTON, J.A., MOLEY, J.F., GREEN, M.V., CARSON, R.E. & MORRISON, S.D. (1985). Parabiotic transfer of cancer anorexia/cachexia in male rats. *Cancer Res.*, **45**, 5547.

OLIFF, A., DEFEO-JONES, D., BOYER, M., MARTINEZ, D. & 4 others (1987). Tumors secreting human TNF/cachectin induce cachexia in mice. *Cell*, **50**, 555.

PONTREMOLI, S. & MELLONI, E. (1986). Extralysosomal protein degradation. *Ann. Rev. Biochem.*, **55**, 455.

RODEMANN, H.P. & GOLDBERG, A.L. (1982). Arachidonic Acid, Prostaglandon $E_2$ and $F_2$ alpha influence rates of protein turnover in skeletal and cardic muscle. *J. Biol. Chem.*, **257**, 1632.

ROFE, A.M., CONYERS, R.A., BAIS, R., GAMBLE, J.R. & VADAS, A.M. (1987). The effects of recombinant tumor necrosis factor (cachectin) on metabolism in isolated rat adipocyte, hepatocyte and muscle preparation (1987). *Biochem. J.*, **247**, 789.

SEMB, H., PETERSON, J., TAVERNIER, J. & 3 others (1987). Multiple effects of tumor necrosis on lipoprotein lipase *in vivo*. *J. Biol. Chem.*, **262**, 8390.

SPRIGGS, D., IMAMURA, K., RODRIGUES, C., HORIGUCHI, J. & KUFE, D.W. (1987). Induction of tumor necrosis factor expression and resistance in a human breast tumor cell line. *Proc. Natl Acad. Sci. USA.*, **84**, 6563.

STOVROFF, M., FRAKER, D.L., SWEDENBORG, J.A. & NORTON, J.A. (1988). Cachechtin/Tumor necrosis factor: a possible mediator of cancer anorexia in the rat. *Cancer Res.*, **48**, 4567.

THEOLOGIDES, A. (1979). Cancer Cachexia. *Cancer*, **43**, 2004.

TRACEY, K.J., WEI, H., MANOGUE, K.R., FONG, Y. & 6 others (1988). Cachectin/tumor necrosis factor induces cachexia, anemia, and inflammation. *J. Exp. Med.*, **167**, 1211.

WAALKES, T.P. & UDENFRIEND, S.A. (1957). A fluorometric method for the estimation of tyrosine in plasma and tissue. *J. Lab. Clin. Med.*, **50**, 733.

WALLACH, D., HOLTMANN, H., ENGELMANN, H. & NOPHAR, Y. (1988). Sensitization and desensitization to lethal effects of tumor necrosis factor and IL-1. *J. Immunol.*, **140**, 2994.

WARREN, R.S., STARNES, H.F., GABRILOVE, J.L., OETTGEN, H.F. & BRENNAN, M.F. (1987). The acute metabolic effects of tumor necrosis factor administration in humans. *Arch. Surg.*, **122**, 1396.